# Validity and reliability of the toxic leadership behaviors of nurse managers scale among Chinese nurses

**DOI:** 10.3389/fpsyg.2024.1363792

**Published:** 2024-03-25

**Authors:** Yumei Zhou, Juanjuan Lin, Xing Liu, Shuping Gao, Fang Yang, Huili Xu

**Affiliations:** Department of Nursing, Xiangyang No.1 People's Hospital, Hubei University of Medicine, Xiangyang, China

**Keywords:** nurse managers, reliability, scale, toxic leadership behaviors, validity

## Abstract

**Objectives:**

Toxic leadership is increasingly becoming common in the nursing field, but the measurement tools are lacking. Therefore, this study aimed to translate the toxic leadership behaviors of nurse managers (ToxBH-NM) scale into Chinese and test its psychometric properties among Chinese nurses.

**Methods:**

The data for this study were obtained from a cross-sectional survey of 1,195 nurses. Exploratory factor analysis (EFA) and confirmatory factor analysis (CFA) were used to examine the structural validity of the ToxBH-NM. The following psychometric properties of the scale were assessed: content validity, criterion validity, internal consistency reliability, and test–retest reliability.

**Results:**

The Chinese version of the ToxBH-NM (C-ToxBH-NM) scale had two dimensions and 30 items. The correlation coefficients between the scores of each item and the total scores were 0762–0.922 (*p* < 0.001), and the range of the CR determination values of all the items were 8.610–18.998, with statistical significance (*p* < 0.001). The total content validity index (CVI) was 0.996, the average CVI was 0.996, and the item-level CVI was 0.875–1.000. Two common factors were identified in the EFA, and 81.074% of the variation was explained cumulatively. The CFA showed that all the fitting indexes reached the standard, and the model fit degree was good. When the Chinese version of the Destructive Leadership Scale was used as calibration, the correlation coefficient was 0.378 (*p* < 0.001). The Cronbach’s alpha coefficients of the overall scale were 0.989 and of the two dimensions were 0.969 and 0.987, respectively, with a split-half reliability of 0.966 and test–retest reliability of 0.978.

**Conclusion:**

The research results show that the C-ToxBH-NM scale has good reliability and validity and can be used to evaluate the severity of toxic leadership behavior among nursing managers.

## 1 Introduction

The current competitive nature of the healthcare industry requires that people are attracted and motivated to participate in leadership roles to meet consumer needs and expectations. In nursing, effective leadership is at the core of management roles, and ample evidence has shown that effective leadership achieves good results among nurses, patients and their families, and organizations (Andrews et al., 2012; Fontes et al., 2019).

To date, most substantial surveys have mainly focused on the positive and effective aspects of leadership, whereas the evaluation of ineffective and negative leadership has mostly been ignored (Rosenstein, 2011). Many leaders in many institutions exhibit toxic leadership behaviors and intentionally or unintentionally harm employees and the organizations they work for Roter (2017). Toxic leadership behaviors, defined as a form of supervision where leaders engage in organized, systematic, and sustained destructive behavior that may cause harm to the entire organization (Webster et al., 2016), Behaviors that indicate negative leadership styles include bullying, jealousy, micromanagement, unfair treatment, narcissism, unethical behavior, authoritarian behavior, distrust of others, aggression, intimidation, manipulation of others, and incompetence (Green, 2014; Bakkal et al., 2019; Milosevic et al., 2020).

In recent years, toxic leadership has become increasingly prominent and widespread in the nursing and other healthcare fields. Existing evidence suggests that continuous exposure to toxic nurse managers or leaders may weaken nurses’ motivation and efforts, possibly leading to adverse work consequences such as job dissatisfaction, job disengagement, poor job performance, job burnout, and frequent absenteeism (Rodwell et al., 2014; Erkutlu and Chafra, 2017; Mullen et al., 2018; Örgev and Demir, 2019). In addition, nurses who work under toxic nurse managers or leaders have reported their inclination to leave their organization and the nursing industry (Lavoie-Tremblay et al., 2016). Employee turnover has significant financial implications for healthcare institutions, as training new nurses is more time and capital intensive (Roche et al., 2015). Therefore, urgent development of a nurse retention strategy, which is currently a top priority, is needed. Furthermore, recognizing the level of toxic behavior among nursing managers and identifying influencing factors are necessary in the development of prevention strategies.

Labrague et al. developed the Toxic Leadership Behaviors of Nurse Managers (ToxBH-NM) scale, which specifically measures toxic leadership behaviors among nurse managers (Labrague et al., 2020). This scale is divided into four behavioral dimensions: intemperate, narcissistic, self-promoting, and humiliating. At the same time, it also reflects the four aspects of toxic leadership, which are supported by a large amount of literature (Green, 2014; Bakkal et al., 2019; Milosevic et al., 2020). The items included in “extremist behavior” are related to the aggressive behavior of nursing managers, reflecting a lack of emotional control. The items included in “narcissistic behavior” are related to behaviors or behaviors exhibited by nursing managers for their own interests or personal agendas. The self-promoting behavior refers to the behavior or actions exhibited by nursing managers to promote personal or professional growth and progress while ignoring the welfare of employees or organizations. Finally, the humiliating behavior reflects the behavior that nursing managers may bring embarrassment and shame to employees (Labrague et al., 2020). This scale has been widely adapted and used in various languages and cultures (Labrague et al., 2020, 2021; Ofei et al., 2022; Farghaly Abdelaliem and Abou Zeid, 2023), but its reliability and validity have only been tested among nurses in Türkiye. Its EFA results show that the scale has four dimensions (Celebi Cakiroglu and Tuncer, 2024), the same as the four dimensions of the original scale (Labrague et al., 2020)^.^ However, the ToxBH-NM scale is rarely used in Chinese settings because it has not yet been translated into Chinese. In China, studies that have evaluated^,^ toxic leadership behaviors among nurse managers have used scales such as the “Abusive Supervision Item” and “Destructive Leadership Scale,” (DLS) which have been validated in China (Tepper, 2000; Zhong, 2013). However, the items in these scales may not accurately reflect the complex nature of the nursing profession.

This study aimed to translate the ToxBH-NM scale from English into Chinese and test its psychometric properties among Chinese nurses. The results of this study can possibly enhance the cross-cultural validation of the scale. In addition, the Chinese version of the ToxBH-NM (C-ToxBH-NM) scale can provide an innovative, a multidimensional, and an effective research tool for related research.

## 2 Methods

### 2.1 Study design

A two-stage cross-sectional study was conducted at a tertiary hospital in China.

Stage I involved translation, retroversion, expert consultation, and pilot testing to assess the content validity.

Stage II involved evaluation of the psychometric characteristics of the translated questionnaire.

This study was reported according to the Strengthening the Reporting of Observational Studies in Epidemiology guidelines (Vandenbroucke et al., 2007).

### 2.2 Stage 1: translation of the original scale from English to Chinese

**Figure 1:**
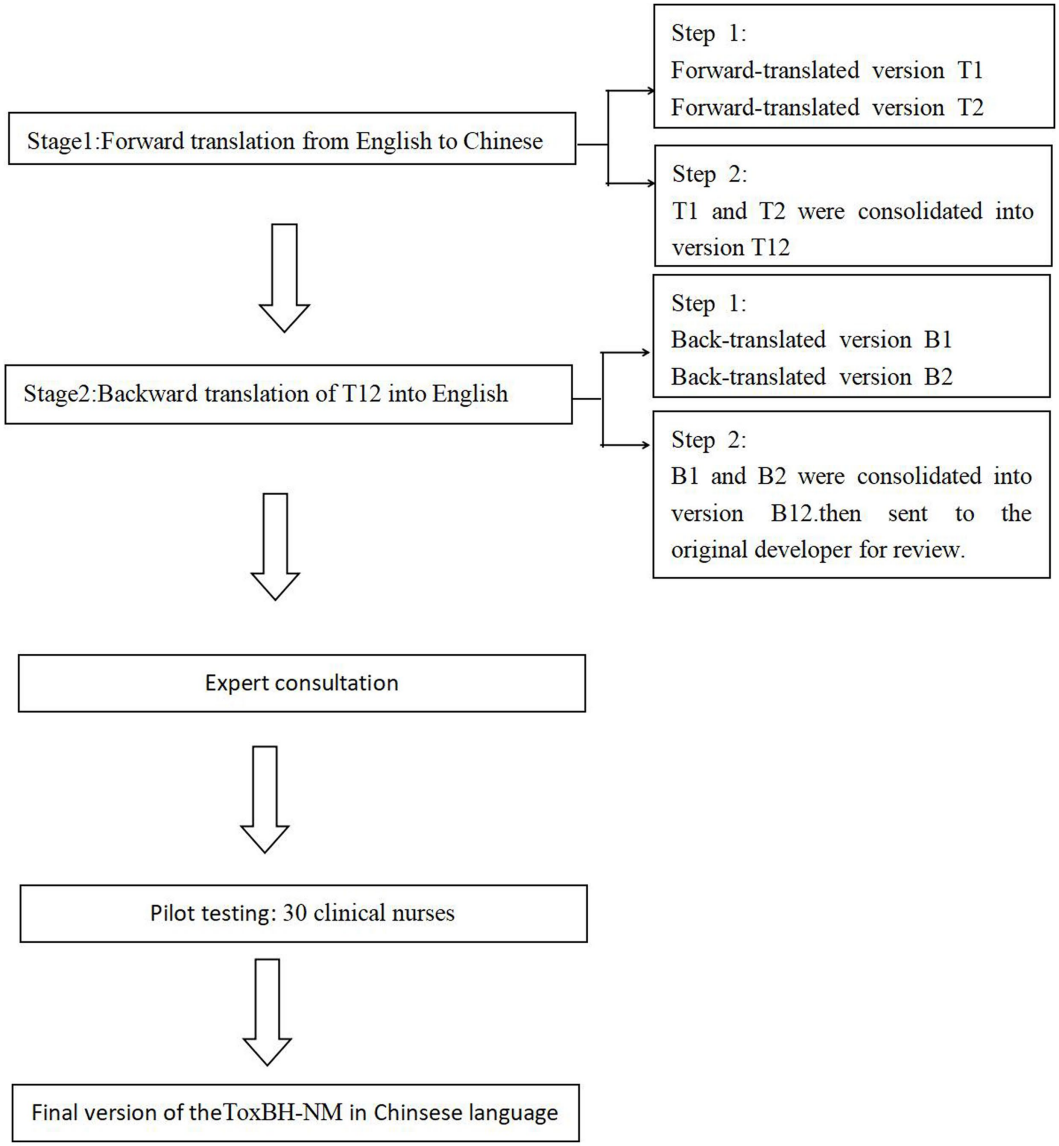
The translation process.

After obtaining authorization from the original author by email, the scale was translated into Chinese following the Brislin’s back-translation model (Brislin, 1970) as shown in Figure 1.

#### 2.2.1 Translation of the ToxBH-NM scale

First, A Doctor of Nursing and a Doctor of Medicine with overseas study experience separately translated the ToxBH-NM scale into Chinese, and then the two had discussions to finalize the C-ToxBH-NM scale.

Second, two other researchers who had not been exposed to the original scale (one with a Master’s degree in English and the other with a Master’s degree in Nursing) back-translated the C-ToxBH-NM scale into English. After both researchers resolved the differences by discussions, the final English version was sent to the original author. The C-ToxBH-NM scale was modified after all language differences were resolved.

#### 2.2.2 Expert consultation

Eight experts from school institutions and tertiary hospitals (2 nursing management experts, 2 nursing clinical experts, 1 nursing education expert, 1 psychology expert, 1 medical management expert, and 1 medical clinical expert) were invited to form an expert committee. The expert group evaluated and modified the C-ToxBH-NM scale in terms of semantics, language expression habits, and professionalism and assessed its content validity.

#### 2.2.3 Pilot testing

The researchers administered the C-ToxBH-NM scale to 30 clinical nurses who were recruited by convenience sampling. The nurses were asked whether the semantics were ambiguous, the items were easy to understand, and the scale was consistent with the domestic cultural background. The total time used to fill out the questionnaire was recorded. The data from this preliminary survey were not included in the formal reliability and validity tests.

### 2.3 Stage 2: psychometric evaluation

#### 2.3.1 Participants

The participants were recruited from a comprehensive hospital in Xiangyang, Hubei Province, China, by convenience sampling. They were registered nurses employed as full-time or part-time hospital staff for more than 1 year. Nurses working in health centers and academic institutions as well as those with less than 1 year work experience were excluded. A total of 1,211 eligible nurses agreed to participate; however, 16 participants were excluded because their questionnaire responses were incomplete, yielding a valid sample size of 1,195 (98.68%). All the participants signed informed consent forms, and this study was approved by the Ethics Committee of Xiangyang No.1 People’s Hospital, Hubei University of Medicine (approval number: 2023KY005).

#### 2.3.2 Instruments

##### 2.3.2.1 Sociodemographic data

Sociodemographic characteristics included gender, age, education level, marital status, department, title, and working years.

##### 2.3.2.2 The ToxBH-NM scale

The original ToxBH-NM scale was developed in 2020 by Labrague et al. (2020) based on the concept of toxic leadership, specifically for evaluating the level of toxic leadership behaviors among nursing managers. The scale includes the following four dimensions: paranoid behavior (15 items), narcissistic behavior (9 items), self-promoting behavior (3 items), and humiliating behavior (3 items), totaling 30 items. Each item is scored on a 5-item Likert scale, ranging from “completely absent” (score of 1) to “frequent” (score of 5), with total scores ranging from 30 to 150 points. A score of 30–69 points indicates mild toxic behavior, 70–110 points indicates moderate toxic behavior, and 111–150 points indicates severe toxic behavior. The Cronbach’s alpha coefficient of the scale was 0.975 and that of each dimension ranged from 0.895 to 0.965. The content validity was 0.957.

##### 2.3.2.3 Chinese version of the destructive leadership scale

This scale was developed by Aasland et al. (2010) based on the destructive leadership model to investigate the frequency of this behavior experienced by research participants over the past 6 months. After Zhong (2013) translated the DLS into Chinese and revised it, the scale included the following three dimensions: authoritarian selfishness (12 items), supportive disloyalty (3 items), and constructive (6 items), with a total of 21 items. Each item was scored on a 4-item Likert scale, with options including “never like this” (score of 1), “sometimes like this” (score of 2), “often like this” (score of 3) and “almost always like this” (score of 4) and with a total score of 21–84 points. The higher the score in each dimension, the stronger the type of leadership behavior. The Cronbach’s alpha coefficient of the Chinese version of the Disruptive Leadership Scale (C-DLS) was 0.85 and that of each dimension ranged from 0.80 to 0.90.

### 2.4 Data collection

Data collection was conducted from January to March 2023 and from January to February 2024. After obtaining consent from the nursing department, the researcher first introduced the research purpose, research significance, and precautions regarding filling the questionnaire to the head nurse during a meeting, and the survey questionnaire QR code was provided. Then, the head nurse distributed the QR code to the department nurses. The questionnaire star backend can only fill in each IP address once, with a filling time of ≤10 min. The description of the research purpose, research significance, and the filling method were set as mandatory options and were required to be read for 1 min before the questionnaire could be filled. Afterward, the researcher reviewed the content and eliminated filled questionnaires with significant bias.

Participants (*n* = 384) recruited from January to March 2023, constituted sample 1, which was used for conduct exploratory factor analysis (EFA), Concurrent validity and Internal consistency. Participants (*n* = 811) recruited from January to February 2024, constituted sample 2, which was used to conduct confirmatory factor analysis (CFA).

### 2.5 Data analysis

SPSS 25.0 (IBM, Armonk, NY, USA) and AMOS 21.0 (IBM) were used to analyze the data. Descriptive data are presented as median (interquartile range) or frequency (percentage).

The decision value and Pearson’s correlation methods were used for item analysis. (1) Pearson’s correlation coefficient method: The correlation coefficient between the score of each item and the total score of the scale was calculated. If the correlation coefficient was less than 0.4, the item was deleted (Polit and Beck, 2006). (2) Decision value method: The total scores were sorted from the highest to the lowest, with the top 27% of the scores categorized as the high group and the bottom 27% as the low group. Independent sample *t*-tests were used to compare the differences in scores between the two groups. If the difference was not statistically significant (*p* > 0.05), the item was considered as poorly differentiated and was deleted (Wu, 2010).

The content validity was evaluated by the expert committee, and each item was scored using a 4-level Likert scoring method with the following options: “unrelated,” “weakly correlated,” “strongly correlated,” and “highly correlated,” which corresponded to 1, 2, 3, and 4 points, respectively. The item-level, total, and average content validity indexes (CVIs) were calculated as >0.78, >0.80, and > 0.90, respectively, indicating that the content validity of the scale was good (Polit et al., 2007).

The construct validity was measured using exploratory factor analysis (EFA) and confirmatory factor analysis (CFA). EFA was performed using principal component analysis with varimax rotation. Bartlett’s test of sphericity and the Kaiser–Mayer–Olkin (KMO) index (KMO >0.80 and *p* < 0.05) were used to determine whether the data were appropriate for factor analysis. Factor extraction and retention were determined using the following criteria: (1) eigenvalues >1; (2) factor loadings ≥0.4 (Pituch and Stevens, 2015), and (3) the selected factor accounting for 40% or more of the total variation (Watkins, 2018). We employed CFA to further assess construct validity. The following criteria were used to evaluate the fit of the CFA model (Sharkey et al., 2019): chi-square/degrees of freedom (χ^2^/df) between 2 and 5; goodness-of-fit, cumulative fit index (CFI), and incremental fit index (IFI) of ≥0.90; and root mean square approximation error (RMSEA) of ≤0.08.

Concurrent validity was assessed using Spearman’s rank correlation analysis between the C-ToxBH-NM scale and C-DLS, with a correlation coefficient between 0.4 and 0.8 considered acceptable (Streiner et al., 2015).

The internal consistency of the scale was evaluated using Cronbach’s alpha coefficient and corrected item-total correlation, with coefficients greater than 0.7 (Rivière et al., 2002) and 0.4 (Lau et al., 2016), respectively, indicating acceptable internal consistency. The split-half reliability involves dividing the scores of the participants into two halves and calculating the correlation coefficients between the scores of the two halves. If the split-half reliability coefficient is greater than 0.6, the evaluation result is considered reliable. The test–retest reliability was determined using intraclass correlation coefficient (ICC), with an ICC of more than 0.7 indicating that the measure is reliable over time (Mirghafourvand et al., 2020).

## 3 Results

### 3.1 Stage 1: content and face validities

The item CVI of the ToxBH-NM scale ranged from 0.875 to 1. The scale CVI was 0.996. For most of the items of the ToxBH-NM scale, the expert committee reached consensus through consultation. Due to differences in cultural backgrounds, only slight inconsistencies in word selection or sentence structure occurred. The item “My nursing manager speaks negatively about his/her staff to other staff in the workplace.” was changed to “My nursing manager slander employees in public,” “My nurse manager blames staff to save him/herself from shame” was changed to “My nursing manager evade their mistakes by criticizing employees,” and “My nurse manager initiates conflict among his/her staff” was changed to “My nursing manager create conflicts between employees without a justifiable reason.” In the pilot test, each item was accepted and recognized by the 30 clinical nurses, easy to understand, and not ambiguous. The questionnaire could be completed within 5 min.

### 3.2 Stage 2

#### 3.2.1 Sample characteristics

A total of 1,195 participants completed the survey. They belonged to 16 departments including Internal Medicine, Surgery, Intensive care unit, Pediatrics, and Obstetrics and Gynecology. The participants were aged 20–60 (33.44 ± 7.893) years and had worked for 0.5–45 (11.33 ± 8.673) years. Descriptive statistics for the other variables are presented in Table 1.

**Table 1:** Participants’ demographic characteristics (*n* = 384).

Characteristics	Cases, *n* (%)
Gender	
Male	18 (4.7%)
Female	366 (95.3%)
Marital status	
Married	289 (75.3%)
unmarried	88 (22.9%)
divorce	7 (1.8%)
Education	
junior college	28 (7.3%)
undergraduate course	347 (90.4%)
Master	9 (2.3%)
Title	
primary title	195 (50.8%)
Intermediate title	160 (41.7%)
Deputy Senior Professional Title	27 (7.0%)
Senior professional title	2 (0.5%)

#### 3.2.2 Item analysis

The correlation coefficient between the scores of each item and the total score ranged from 0762 to 0.922 (*p* < 0.001) and that between the items ranged from 0.587 to 0.774 (*p* < 0.001), so no item was deleted. Regarding the decision value method, the independent sample *t*-test analysis of the differences between the high and low groups of the nurses was performed. The results showed that the Critical value of all the entries ranged from 8.610 to 18.998, with statistically significant differences between the groups (*p* < 0.001). This indicates that the items in the scale had good discrimination.

#### 3.2.3 Construct validity

The KMO index was 0.976, and Bartlett’s test (*χ*^2^ = 37033.541, *p* < 0.001) indicated that the data were suitable for EFA. Using principal component analysis and the maximum variance rotation method, two common factors with feature roots >1 were extracted, with a cumulative variance contribution rate of 81.074%. All the 30 items have a corresponding factor load greater than 0.5 (Table 2), indicating good structural validity.

**Table 2:** The result of factor analysis of the ToxBH-NM and item-total correlation (*n* = 384).

Items	Factor loading	
	Factor 1	Factor 2
1. My nursing managers slander employees in public	**0.819**	0.371
2. My nurse manager places his/her personal interest ahead of others.	**0.807**	0.436
3. My nurse manager employs in deception to look good to his/her superiors.	**0.805**	0.471
4. My nurse manager believes that the future of the unit or ward only goes well with him/her.	**0.799**	0.444
5. My nurse manager has a group of dedicated staff who implement his/her orders.	**0.794**	0.432
6. My nurse manager cares only for his/her own ward/unit and not with others.	**0.794**	0.432
7. My nurse manager only treats favorably those staff that bears profit for him/her.	**0.789**	0.460
8. My nurse manager believes that he/she is an extraordinary person.	**0.787**	0.462
9. My nurse manager believes that he/she is an extraordinary person.	**0.782**	0.406
10. My nurse manager repeatedly reminds staff of their previous mistakes.	**0.780**	0.467
11. My nurse manager changes his/her behavior swiftly when his/her supervisor is present.	**0.772**	0.463
12. My nurse manager declines to share the accountability of the mistakes which the staff make.	**0.763**	0.451
13. My nurse manager repeatedly reminds his/her staff that they are incompetent and inefficient at work.	**0.755**	0.491
14. My nursing managers create conflicts between employees without a justifiable reason.	**0.745**	0.478
15. My nurse manager does not trust anyone else to complete tasks effectively.	**0.737**	0.419
16. My nurse manager ignores his/her staff as if they do not exist.	**0.730**	0.730
17. My nurse manager thinks he/she is always right.	**0.719**	0.420
18. My nurse manager punishes the entire unit for mistakes made by one staff.	**0.712**	0.320
19. My nurse manager easily gets annoyed when being questioned by her/his staff.	**0.672**	0.328
20. My nurse manager belittles his/her staffs’ work.	**0.632**	0.218
21. My nurse manager believes that he/she deserves the position that he/she is in to the full extent.	**0.600**	0.189
22. My nurse manager raises voice when his/her point is not favored or accepted by staff.	0.282	**0.840**
23. My nurse manager causes staff to try to‘read’his/her temper.	0.477	**0.804**
24. Nursing managers evade their mistakes by criticizing employees	0.484	**0.773**
25. My nurse manager takes a stand against staff without listening to them first.	0.400	**0.765**
26. My nurse manager is rude and disrespectful to staff.	0.378	**0.722**
27. My nurse manager shows arrogance to his/her staff.	0.418	**0.715**
28. My nurse manager allows his/her mood to command the climate of the unit or ward.	0.429	**0.707**
29. My nurse manager disregards ideas of his/her staff that are contrary to his/her own.	0.462	**0.662**
30. My nurse manager does not value his/her staffs’contribution to the organization.	0.381	**0.652**
Eigenvalues	9.929	
Cumulative variance (%)	81.074	

The meaning of the bold values was that the factor loadings were greater than 0.50.

**Figure 2:**
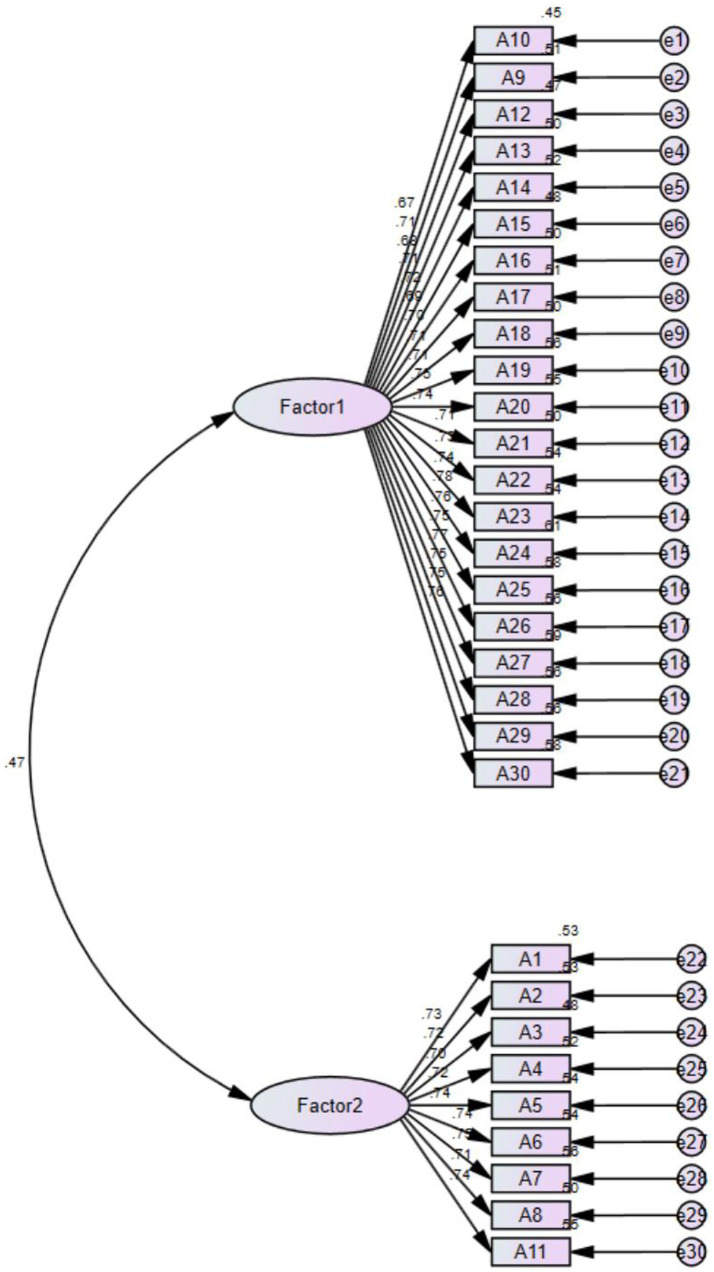
The confirmatory factor analysis of a two-factor model of C-ToxBH-NM.

The CFA showed that the scale meets the measurement standards, and the model has good adaptability and structural validity (χ^2^/df = 2.437, Root mean square approximation error (RMSEA) = 0.042, Norm Fit Index (NFI) = 0.935, Incremental fit index (IFI) = 0.960, Tucker-Lewis Index (TLI) = 0.957, and Comparative Fit Index (CFI) = 0.960). The CFA mode is shown in Figure 2.

#### 3.2.4 Concurrent validity

The correlation coefficients between the C-ToxBH-NM scale and C-DLS were good (*r* = 0.578, *p* < 0 0.001), indicating that the concurrent validity of the C-ToxBH-NM was satisfactory.

#### 3.2.5 Reliability

The Cronbach’s alpha coefficient of the C-ToxBH-NM scale was 0.989 and that for the two dimensions were 0.969 and 0.987, respectively. The ICC was 0.978, and the split-half reliability was 0.966.

## 4 Discussion

This study translated and validated the ToxBH-NM scale in the Chinese context, providing empirical evidence for the psychometric properties of a leadership scale among Chinese nursing managers.

The results of this study show that the content validity of the C-ToxBH-NM scale is good, indicating that ToxBH-NM is scientific and reasonable. In addition, after expert consultation and pilot testing, the scale was found to be clear and suitable for evaluating the leadership behavior of nursing managers. Furthermore, items on the C-ToxBH-NM scale were homogenous, relevant, functional, and not redundant as reflected by the inter-item/inter-total correlations, with inter-item correlations of 0.587 to 0.774 and item-total correlations of 0.762 and 0.922, showing a significant positive correlation (*r* > 0.4). This finding indicates that each item of the C-ToxBH-NM scale can effectively reflect the overall toxic leadership level of nursing managers.

CFA is used to determine the validity of measurement tools developed based on other samples and cultures (Murat, 2017). In this study, the EFA and CFA demonstrated that the construct validity of the scale was good. EFA proved that the scale has only two dimensions, which is different from the four dimensions of the original scale (Labrague et al., 2020). This difference may be related to the race of the research participants. On the one hand, Chinese work culture emphasizes the harmony of collective relationships. Managers need to be impartial and caring for their subordinates. On the other hand, it requires managers to strictly reward and punish and target orientation. There is a significant power distance and clear organizational hierarchy in Chinese work (Sun et al., 2020; Yin et al., 2021). Due to the influence of traditional Chinese culture on the leadership behavior of Chinese managers (Ma and Tsui, 2015), compared to foreign countries, when nursing managers do not like or blame employees, they rarely show obvious suprasocial “hostility” and prefer to use covert and disguised ways to express these thoughts and feelings implicitly (Guo et al., 2022). Therefore, toxic leadership in the context of Chinese culture may differ from other countries. The CFA results indicated that all the fitting data of the C-ToxBH-NM scale are within an acceptable range. Additionally, significant associations between the C-ToxBH-NM scale and C-DLS were observed. These results support the assumption that the C-ToxBH-NM scale has satisfactory criteria-related reliability among Chinese nurse.

The internal consistency of the C-ToxBH-NM scale was acceptable, with a Cronbach’s alpha coefficient of 0.989, which was higher than the generally accepted guideline coefficient of 0.80 (Lance et al., 2006). Compared with the original scale, the Cronbach’s alpha coefficient of the Chinese version is higher than that of the English version.

Finally, the test–retest reliability coefficient of the C-ToxBH-NM was 0.978, indicating that the scale has good reliability over a period. This result provides evidence for the effectiveness of intervention measures aimed at reducing the level of toxic leadership among nursing managers.

### 4.1 Limitations

When interpreting the results of this study, several limitations need to be considered. (1) The researchers conducted investigations in only one tertiary hospital in Xiangyang City. Hospitals in other areas of Xiangyang City were excluded, thereby limiting the generalizability of our research results. Future research should expand the research population and centers. (2) Due to different cultural backgrounds, reaction bias (social design ability bias) could have occurred. This means that respondents hope to perform well in the survey or showcase a good image of their hospital, which may have affected the research results, although the survey was anonymous.

## 5 Conclusion

In recent years, the leadership of nursing managers has become a hot topic with increasing attention both domestically and internationally, but negative leadership is often overlooked, and no specific measurement tools are available. This study strictly followed the principles of translation, back translation, and cultural adjustment based on Brislin’s translation method to translate the ToxBH-NM questionnaire into Chinese. The C-ToxBH-NM scale included two dimensions and a total of 30 items. The scale has a short title, is easy to understand, has moderate items, and requires a filling duration of 5.47 min, indicating strong feasibility. The research results show that the scale has good reliability and validity and can be used to evaluate the severity of toxic leadership behavior among nursing managers. The total score of this scale can be divided into three levels (mild, moderate, and severe) to provide targeted intervention measures. Moreover, this scale can be used to dynamically evaluate the effectiveness of interventions in real time, indicating its good practical value.

## Data availability statement

The original contributions presented in the study are included in the article/supplementary material, further inquiries can be directed to the corresponding author.

## Ethics statement

The studies involving humans were approved by the Ethics Committee of Xiangyang No.1 People’s Hospital, Hubei University of Medicine. The studies were conducted in accordance with the local legislation and institutional requirements. The participants provided their written informed consent to participate in this study.

## Author contributions

YZ: Data curation, Investigation, Project administration, Writing – original draft, Writing – review & editing. JL: Data curation, Writing – original draft, Writing – review & editing. XL: Data curation, Writing – original draft. SG: Data curation, Writing – review & editing. FY: Data curation, Writing – review & editing. HX: Conceptualization, Data curation, Methodology, Supervision, Writing – original draft.
